# Comparing the stigma experiences and comfort with disclosure in Dutch and English populations of people living with dementia

**DOI:** 10.1177/14713012231188503

**Published:** 2023-07-22

**Authors:** Jem Bhatt, Gianna Kohl, Katrina Scior, Georgina Charlesworth, Majon Muller, Rose-Marie Dröes

**Affiliations:** UCL Unit for Stigma Research, Clinical, Educational and Health Psychology, 4919 University College London, London, UK; Research Department of Clinical, Educational and Health Psychology, 4919 University College London, London, UK; UCL Unit for Stigma Research, Clinical, Educational and Health Psychology, 4919 University College London, London, UK; Research Department of Clinical, Educational and Health Psychology, 4919 University College London, London, UK; Research and Development, 26066 North East London Foundation Trust, London, UK; Department of Internal Medicine, Geriatric Medicine Section, Amsterdam Cardiovascular Sciences Institute, Amsterdam University Medical Centers, location VUmc, Amsterdam, The Netherlands; Department of Psychiatry, Amsterdam University Medical Centers, location VUmc/Amsterdam Public Health Research Institute, Amsterdam, The Netherlands

**Keywords:** dementia, disclosure, psychometrics, secrecy, stigma

## Abstract

**Objectives:**

People living with dementia can feel hesitant disclosing their diagnosis to social networks, partly due to stigma. Little attention has been paid to the measurement of disclosure decisions and stigma, and few standardised stigma tools have been validated in languages other than English. We investigated the psychometric properties of Dutch translations of three stigma measures, and explored the stigma experiences of Dutch and English people living with dementia as well as patterns and predictors of comfort with disclosure.

**Methods:**

Community-dwelling adults living with dementia in the Netherlands (*n* = 40) and England (*n* = 40) completed either the English versions or the Dutch translations of the Comfort with Disclosure scale and three stigma measures (Stigma Impact, Stigma Stress, and Secrecy Scale). We established the psychometric properties of the stigma measures and conducted correlation and regression analyses.

**Results:**

Internal consistency was good to excellent for all measures in the Dutch sample. Small but significant differences were found between the Dutch and English samples on the total score of the Stigma Impact Scale and its subscale social isolation. Age was negatively associated with comfort disclosing to family, and desire for secrecy was negatively associated with comfort disclosing to both family and friends.

**Conclusions:**

The psychometric properties of the Dutch scales were satisfactory. Many people living with dementia would feel comfortable disclosing their diagnosis to family and friends, but stigma experiences can greatly affect this decision. Cross-cultural differences in stigma experiences in persons with dementia require further investigation.

## Introduction

Receiving a diagnosis of dementia is a major life event that can have benefits for individuals but can also be a cause for concerns or dilemmas (Low et al., 2019), such as the stigma that is associated with dementia (Nguyen & Li, 2020; Read et al., 2017; Stockwell-Smith et al., 2019). One of the decisions people living with dementia are faced with is if and how they want to disclose their diagnosis to their social networks, how much they want to disclose and at what point in time. Disclosure decisions such as these are often contemplated in the context of stigma-related behaviours, for example, delayed help-seeking and withdrawing from everyday activities or interactions, and reactions, for example, loss of confidence and feeling inferior (Devlin et al., 2006; Morgan et al., 2002; O’Sullivan et al., 2014; Walmsley & McCormack, 2016; Werner et al., 2010). Overall, stigma can be a great source of stress for individuals when they perceive the stigma as harmful and feel they lack the required resources to cope with it, as suggested by the stress-coping model examined by Rüsch et al. (2009). The measurement of stigma and its impact on diagnostic disclosure along with other associated factors among people living with dementia is therefore a priority.

### Self-stigma

Self-stigma, also referred to as internalised stigma, is a cognitive process that occurs when individuals internalise negative stereotypes, prejudice, and discrimination about their stigmatised identity (Corrigan & Watson, 2002). Feelings of self-stigma have been shown to correlate negatively with a range of psychosocial variables such as self-esteem, quality of life, and self-efficacy among people with mental health difficulties (Livingston & Boyd, 2010). Research among people living with dementia has found associations between self-stigma and depression, anxiety, self-esteem, perceived social support, activity participation, and physical health (Burgener et al., 2015a; Burgener, Buckwalter, Perkhounkova, Liu, et al., 2015b). Consequences of self-stigma, in turn, can be far-reaching and may include the aforementioned behaviours (e.g., withdrawing from activities or delayed help-seeking) and reactions (e.g., loss of confidence).

### Measuring self-stigma

The Stigma Impact Scale is a psychometrically robust instrument for use with English-speaking people living with dementia, measuring perceived self-stigma or the impact stigma has on the self (Burgener & Berger, 2008). It considers four consequences of self-stigma: social rejection, internalised shame, social isolation, and financial insecurity. Recent testing of this scale in the United Kingdom (UK) suggests an association between lower self-esteem and higher internalised shame and social isolation (Bhatt et al., 2021), confirming the inverse relationship between self-esteem and stigma concepts found in other populations (Corrigan et al., 2015; Kaiser et al., 2004). The Stigma Impact Scale has been translated into Polish and Italian for use in the evaluation of the Meeting Centres Support Programme (MEETINGDEM; Mangiaracina et al., 2017; Szcześniak et al., 2017). However, although this programme originated in the Netherlands, data have not, as yet, been collected on stigma impact with Dutch people living with dementia. Therefore, validating the Stigma Impact Scale in a Dutch sample is necessary.

### Diagnostic disclosure and stigma

Whereas historically a diagnosis of dementia was often kept secret from individuals with the condition, current international dementia strategies as well as the global action plan by the World Health Organization have set out measures that aim for the early detection and timely diagnosis of dementia (Alzheimer Europe, 2018; World Health Organization, 2017). In a worldwide survey, a quarter of people living with dementia and a third of informal carers reported hiding a diagnosis of dementia because of stigma (Alzheimer’s Disease International, 2019). Secrecy about one’s diagnosis, a term associated with Link et al.’s (2002) research on individuals’ responses to stigma and rejection, can lead to social withdrawal (Camacho et al., 2020), in turn associated with increased risk of cognitive decline (Lara et al., 2019). The decision to conceal or reveal a diagnosis of dementia can be a source of stress and discomfort for people living with dementia and their immediate family, but factors associated with disclosure-related comfort are not well understood. For other stigmatised conditions, there is a growing body of evidence suggesting an association between perceived stigma and the perceived need to be secretive about one’s diagnosis or health condition (Benoit et al., 2018; Corrigan et al., 2016; Oexle et al., 2017; Thornicroft et al., 2016), in addition to the experience of stigma-related harm and reduced ability to cope (Mulfinger et al., 2018; Rüsch et al., 2009). However, these concepts have not yet been given appropriate attention in the dementia field.

### Aims and rationale

The current study was designed in response to a lack of understanding of people living with dementia’s comfort with diagnosis disclosure and an absence of a Dutch self-stigma measure. More specifically, the aims of this study were to (1) establish the acceptability, reliability, and validity of the Stigma Impact Scale and other stigma measures with a Dutch sample, (2) understand differences in outcome measures of stigma between Dutch and English people living with dementia, (3) explore the relationships between disclosure comfort, stigma impact, stigma-related harm, resources to cope, and secrecy in a Dutch and English sample, and (4) establish factors associated with disclosure comfort.

## Methods

### Design

A cross-sectional interview design was used to test the reliability and validity of the Stigma Impact Scale and related constructs in a Dutch population, and to assess the relationship and possible differences between the different constructs in people living with dementia in the Netherlands and England. A power analysis indicated that with a sample of 40 each for Dutch and English participants, medium to large differences between the two groups could be demonstrated with a power of .90 and a Cronbach’s alpha of .05.

### Participants

We recruited convenience samples in the Netherlands and England. In the Netherlands, recruitment took place via several meeting centres (Dröes et al., 2011) for people living with dementia and their family carers and the Centre of Geriatric Medicine Amsterdam of the Amsterdam University Medical Center, location VUmc. In England, participants were recruited via the Join Dementia Research Database, study advertisements relying on self-identification, and outreach activities organised by the Alzheimer’s Society UK. Eligibility criteria required participants to be over 18 years of age with a primary progressive diagnosis of dementia. We excluded participants if they had significant sensory impairments precluding participation and if they lacked capacity to consent to study participation. Eligibility criteria were consistent across countries. Ethical approval was granted by the UCL Research Ethics Committee (registration number: 11501/002). The medical ethics committee of the VU University Medical Center declared this study exempt from the Medical Research Involving Human Subjects Act. All participants received written and oral information about the study.

### Measures

Sociodemographic details and information related to the dementia diagnosis included age, gender, ethnicity, employment status, living situation, first language, type of dementia, months since diagnosis, and, only in the Netherlands, severity of dementia and education. Severity of dementia was assessed using the Mini-Mental State Examination (Folstein et al., 1975) or, only in the Netherlands, the Global Deterioration Scale (Reisberg et al., 1982).

The Comfort with Disclosure Scale (Mulfinger et al., 2018; Rüsch et al., 2014) was used to asses comfort with disclosing the dementia diagnosis to two disclosure targets: family (‘In general, how comfortable would you feel talking to a family member about dementia, for example, telling them you have a dementia diagnosis and how it affects you?’) and a friend (same item but ‘family’ replaced with ‘friend’). Each item was rated by participants from one (‘not at all’) to seven (‘very much’), with a midpoint of four (‘moderately’). A higher score indicates more disclosure comfort.

The Stigma Impact Scale (Burgener & Berger, 2008) comprises 21 items measuring self-stigma with three subscales: social rejection (nine items, e.g., “I feel others avoid me because of my impairment”), internalised shame (five items; e.g., “I feel others think I am to blame for my impairment”) and social isolation (seven items; “I feel set apart from others who are well”). Items were rated on a scale of one (‘strongly disagree’) to four (‘strongly agree’), with an option of zero for ‘not applicable’. Consistent with previous research conducted with people living with dementia, we excluded the financial security subscale (Lion et al., 2020; Mangiaracina et al., 2017; Szcześniak et al., 2017). We calculated the scale’s total score by summing the scores of all items; this process was also followed for each subscale. The internal consistencies of the Stigma Impact Scale subscales reported in the MEETINGDEM project ranged between the three countries from .65-.82 for social rejection, .69–.80 for internalised shame, and .67–.84 for social isolation (Lion et al., 2020).

The Stigma Stress Scale (Kaiser et al., 2004; Rüsch et al., 2009) was used to measure the perceived harm caused by stigma (four items; e.g., ‘Stigma against people living with dementia will affect many areas of my life’) and the resources to cope with stigma (four items; e.g., ‘I am prepared to deal with stigma against people living with dementia’). Each item is rated from one (‘strongly disagree’) to seven (‘strongly agree’). Responses are summed, giving a possible total score of 4 to 28 for each subscale, a higher score indicating more perceived stigma-related harm and resources to cope with stigma. For the English language version for people living with dementia, Cronbach’s alpha was .94 for the perceived stigma-related harm subscale and .87 for the resources to cope subscale (Bhatt et al., 2021).

The Secrecy scale of the Stigma Coping Orientation Scale (Link et al., 2002) consists of seven items rated by participants from one (‘strongly disagree’) to four (‘strongly agree’), assessing the extent to which concealment as a means of avoiding stigmatisation and rejection from others is endorsed (e.g., ‘In view of society’s negative attitudes towards people living with dementia, you would advise people with dementia to keep it a secret’). We calculated the total score by taking the overall mean of the measure, a higher mean score indicating more secrecy. Previous research with the English version reported adequate internal consistency with a Cronbach’s alpha of .87 (Bhatt et al., 2021).

The 10-item Rosenberg Self-Esteem scale (Rosenberg, 1965) was used to measure self-esteem (e.g., “On the whole, I am satisfied with myself”). Items are rated from one (‘strongly disagree’) to four (‘strongly agree’), with the total score ranging from 10 to 40. We used the Rosenberg Self-Esteem Scale to establish the convergent validity of the stigma measures as previous research has reported an inverse relationship between self-esteem and stigma (Burgener & Berger, 2008).

### Procedure

Potential participants received written study information and were individually informed of the aim and procedure of the study by the researchers, either in person or by telephone. In the Netherlands, data were collected at meeting centres in the Amsterdam region or in the Centre of Geriatric Medicine Amsterdam. In England, data were collected online using the platform Qualtrics or face to face in participants’ homes. All data were collected by trained interviewers and all participants provided written consent. Completion of the measures lasted between 35 and 120 minutes, with a mean of 44 minutes.

### Data analysis

Statistical analyses were performed using the software IBM SPSS Statistics 25.0. Missing values and normality checks were performed on all variables. (1) The acceptability, reliability, and validity of the Stigma Impact Scale and other existing stigma measures in the Dutch sample were measured using completion rates, Cronbach’s alphas for internal consistency, and Pearson correlations coefficients for convergent validity of stigma measures and the Rosenberg Self-Esteem Scale. (2) Differences in the characteristics between the Dutch and English samples were determined using Chi-squared tests for categorical variables and Mann-Whitney *U* test for continuous variables. To understand the differences in stigma between the Dutch and English analyses, analyses of co-variance (ANCOVAs) were used. For each measure, effect sizes, i.e. partial eta squared ( $\eta_{p}^{2}$ ), were calculated. An effect size of .01 was interpreted as small, .06 as medium, and 0.14 as large (Cohen, 1988). (3) The relationships between comfort with disclosure, stigma impact, secrecy, and resources to cope in the Dutch and English sample were explored through correlations. Correlations of .10–.30 were interpreted as weak, correlations of .40–.60 as moderate, and correlations of .70–.90 as strong (Dancey & Reidy, 2017). (4) Ordinal regression analyses were carried out to establish predictors of comfort with disclosure. Variables were entered into the model based on the outcomes of the correlations. The assumptions of no multicollinearity and proportional odds were tested and met. All tests were conducted with a significance level of alpha of 0.05.

## Results

### Participant characteristics

Eighty participants took part in this study, forty from the Netherlands and forty from England (see Table 1). The majority of participants identified as ‘white’ (93%) and had Alzheimer’s disease (59%), followed by vascular dementia (13%) and mixed dementia (13%). In the Dutch sample, education and stage of dementia were also assessed, with the majority having completed high school only (50%) and having mild to moderate dementia (60%). There were significant differences in ‘age’ and ‘time since diagnosis’ between the two samples, with all Dutch participants being over the age of 69 whereas 11 participants in the English sample were under the age of 65. The average time since diagnosis was more than twice as long for English participants compared to their Dutch counterparts. No differences were found between the samples in terms of ethnicity, gender, cohabitation or employment status.

**Table 1.** Participant characteristics of the Dutch (*n* = 40) and English (*n* = 40) samples.

Participant characteristics		Dutch, *M* (*SD*) or *n*	English, *M* (*SD*) or *n*	Test for difference/association
Age in years, min-max		80.9 (4.8), 69-90	72.4 (10.6), 56-95	*U* = 403.500, *p* =.000
Months since diagnosis, min-max		21.4 (16.3), 2-72	45.2 (33.1), 2-120	*U* = 451.000, *p* =.001
Gender (M/F)		16/24	20/20	*x*^2^(1) = 0.81, *p* = .369
Type of diagnosis	Alzheimer’s disease	26	21	*x*^2^(6) = 8.49, *p* = .205
	Vascular dementia	3	7	
	FTD	0	1	
	Lewy body dementia	3	1	
	Mixed dementia	4	6	
	ND/Unknown	4	4	
Ethnicity	White	38	36	*x*^2^(3) = 5.04, *p* = .169
	Other ethnic group	2	3	
	ND	0	1	
Living alone	Yes	18	13	*x*^2^(1) = 1.32, *p* = .251
	No	22	27	
Education	Less than high school	4	Not available	
	High school	20		
	MBO	4		
	HBO/Bachelor’s	7		
	Master’s	3		
	PhD	2		
Severity of dementia^a^	Very mild	13	Not available	
	Mild	11		
	Moderate	14		
	Severe	1		
Employment status	Employed	1	5	*x*^2^(3) = 5.07, *p* = .167
	Retired	37	30	
	ND/Other	2	5	
First language	Dutch	37	0	
	English	0	37	
	Other	3	3	

*Note*. FTD = Frontotemporal dementia; HBO = Hoger beroepsonderwijs/Higher education; MBO = Middelbaar beroepsonderwijs/Vocational training; ND = Not disclosed.

^a^Using the Mini-Mental State Examination.

### Acceptability, reliability and validity of the Stigma Impact Scale and other stigma measures in a Dutch sample

There were no significant violations of normality with the exception of the resources to cope subscale of the Stigma Stress Scale. Fewer than 15% of participants recorded the lowest or highest scores, indicating no floor or ceiling effects. All stigma measures had adequate internal consistency (Cronbach’s alpha scores ≥ .70; see Table 2) with the exception of the internalised shame subscale of the Stigma Impact Scale (α = .62).

**Table 2.** Data distributions and psychometric properties of stigma measures in the Dutch sample (*n* = 40).

Stigma variables	Floor and ceiling					
	Skewness	Kurtosis	Lowest score (%)	Highest score (%)	Internal consistency (α)	Convergent validity Self-esteem
Stigma impact Scale						
Total	0.59	−0.15	0	0	.89	−.58**
Social rejection	1.10	1.28	0	0	.84	−.44**
Internalised shame	0.49	0.23	0	0	.52	−.43**
Social isolation	0.33	−0.90	0	0	.76	−.57**
Stigma Stress Scale						
Stigma-related harm	0.22	−1.11	10.0	2.5	.92	−.48**
Resources to cope	−1.35	2.50	0	5.0	.73	−.33* ^
Secrecy	−0.15	−0.45	10.0	0	.84	−.33*

^ Non-parametric Spearman’s rank correlations.

* Correlation is significant at the 0.05 level; **Correlation is significant at the 0.01 level.

Convergent validity with self-esteem was established for all stigma measures in the Dutch sample (see Table 2). There were significant moderate negative correlations between stigma impact and self-esteem, and significant weak to moderate correlations between perceived stigma-related harm, resources to cope, secrecy, and self-esteem. The directionality of the correlations was as hypothesised.

### Differences in outcome measures between the Dutch and English sample

Differences between the two samples on the variables were established using ANCOVA (see Table 3). The variables ‘age’ and ‘time since diagnosis’ were included as covariates as these differed between the two samples. There were significant differences between the two groups on the total Stigma Impact Scale (*F*(1,80) = 4.14, *p* = .045, $\eta_{p}^{2}$ = .052), with English participants experiencing more self-stigma (*M* = 39.92, 95% confidence interval [CI] = 36.48–43.35) than participants from the Netherlands (*M* = 34.52, 95% CI = 31.08–37.95). Based on the estimated marginal means, the covariate of age accounted for a greater proportion of the variance in the Stigma Impact Scale total score (*F*(1,80) = 13.71 *p* < .001, $\eta_{p}^{2}$ = .153) than did time since diagnosis (*F*(1,80) = 1.05, *p* > .05, $\eta_{p}^{2}$ = .014).

**Table 3.** Means for all scales in the combined sample (*N* = 80) including results of the ANCOVA and effect sizes.

Variables	Unadjusted means		Adjusted means^a^		ANCOVA^a^	η_p_^2^
	Dutch, *M* (*SD*)	English, *M* (*SD*)	Dutch, *M* (*SD*)	English, *M* (*SD*)	Test statistic	
Comfort with disclosure to family	4.88 (1.90)	5.88 (1.56)	5.00 (1.92)	5.75 (1.92)	*F*(1,80) = 2.60, *p* = .111	.033
Comfort with disclosure to friends	5.00 (1.62)	5.55 (1.83)	5.10 (1.93)	5.45 (1.93)	*F*(1,80) = 0.53, *p* = .469	.007
Stigma impact Scale						
Total	31.88 (7.72)	42.55 (12.88)	34.52 (10.90)	39.92 (10.90)	*F*(1,80) = 4.13, *p* = .045	.052
Social rejection	12.20 (3.78)	17.25 (6.91)	13.64 (5.71)	15.81 (5.71)	*F*(1,80) = 2.42, *p* = .124	.031
Internalised shame	7.62 (1.93)	8.60 (2.84)	7.65 (2.66)	8.57 (2.66)	*F*(1,80) = 2.01, *p* = .161	.026
Social isolation	12.06 (3.47)	16.70 (5.53)	13.22 (4.78)	15.54 (4.78)	*F*(1,80) = 3.98, *p* = .050	.050
Stigma Stress Scale						
Stigma-related harm	14.21 (6.75)	15.73 (8.16)	14.14 (7.98)	15.80 (7.98)	*F*(1,80) = 0.73, *p* = .394	.010
Resources to cope	22.15 (3.93)	23.48 (4.02)	22.18 (4.43)	23.44 (4.43)	*F*(1,80) = 1.35, *p* = .249	.017
Secrecy	1.79 (0.46)	1.84 (0.64)	1.72 (0.61)	1.90 (0.61)	*F*(1,80) = 1.45, *p* = .232	.019
Self-esteem	32.63 (4.09)	29.68 (4.55)	31.99 (4.74	30.31 (4.74)	*F*(1,80) = 2.13, *p* = .148	.027

^a^Controlled for ‘age’ and ‘time since diagnosis’.

The difference in the Stigma Impact Scale total score was mainly due to a significant difference in the Stigma Impact Scale social isolation subscale (*F*(1,80) = 3.98, *p* = .05, $\eta_{p}^{2}$ =.050), with English participants experiencing higher levels of social isolation (*M* = 15.5, 95% CI = 14.04–17.04) than Dutch participants (*M* = 13.2, 95% CI = 11.72–14.72). The effect size was large. As for the Stigma Impact Scale total score, the covariate of age (*F*(1,80) = 11.20 *p* < .001, $\eta_{p}^{2}$ = .128) accounted for a greater proportion of the variance in social isolation sores than did time since diagnosis (*F*(1,80) = 2.09, *p* < .05, $\eta_{p}^{2}$ = .027). No significant differences between the Dutch and English participants were found on any other variables.

### Relationship between comfort with disclosure and outcome variables

Descriptive statistics and visually inspecting the histograms determined that the Comfort with Disclosure Scale was not normally distributed; comfort with disclosure to family and to friends scores were negatively skewed (both −1.06; see Table 4 for the data distributions).

**Table 4.** Frequencies of comfort with disclosure for the combined sample (*N* = 80).

Disclosure target	Degree of comfort with disclosure, *n* (%)							Comfort with disclosure score	
	Not at all	A little	Somewhat	Moderately	Considerably	A great deal	Very much	Mean	*SD*
Family	4 (5)	5 (6.25)	4 (5)	7 (8.75)	13 (16.25)	18 (22.5)	29 (36.25)	5.4	1.80
Friends	5 (6.25)	4 (5)	1 (1.25)	9 (11.25)	21 (26.25)	15 (18.75)	25 (31.25)	5.3	1.74

The majority of the 80 participants would generally feel comfortable disclosing their diagnosis to family and friends: 61 participants would feel considerably to very comfortable disclosing to family, and 60 participants would feel considerably to very comfortable disclosing to friends. The degree of comfort with disclosure was quite consistent across the two disclosure recipient categories, with slightly higher levels of disclosure comfort reported to family (*M* = 5.4, *SD* = −1.80) than friends (*M* = 5.3, *SD* = −1.74). The correlations between comfort with disclosure and outcome variables are summarised in Table 5.

**Table 5.** Relationship between comfort with disclosure and variables for the combined sample (*N* = 80).

	Self-esteem	Stigma-related harm	Resources to cope	Secrecy	Social rejection	Internalised shame	Social isolation	Age	Time since diagnosis	Comfort with disclosure to family
Stigma-related harm	−.42**									
Resources to cope	.23*^	−.31**^								
Secrecy	−.18	.18	−.17^							
Social rejection	−.50**^	.50**^	−.17^	.03^						
Internalised shame	−.43**	.25*	−.09^	.45**	.42**^					
Social isolation	−.62**	.56**	−.19^	.03	.74**^	.40**				
Age	.31**	−.29**	−.05^	.18	−.44**^	−.23*	−.50**			
Time since diagnosis	−.19^	.22^	.16^	−.07^	.31**^	−.07^	.23*^	−.19^		
Comfort with disclosure to family	.05^	−.12	.27*	−.40**	.15	−.25*	.05	−.33**^	.10^	
Comfort with disclosure to friends	.13^	−.25*	.26*	−.58**	.14	−.36*	−.03	−.21^	.13^	.70**^

* Correlation is significant at the 0.05 level; **Correlation is significant at the 0.01 level.

^ Non-parametric Spearman’s rank correlations.

### Predictors of comfort with disclosure

The results of the ordinal regression analyses investigating predictors of comfort with disclosure to family and friends are displayed in Table 6. For comfort with disclosure to family, the model contained the predictors age, resources to cope with stigma, secrecy, and internalised shame. Age and secrecy as a means to avoid stigma were significantly associated with disclosure comfort toward family. Being older was associated with a decrease in the odds of comfort disclosing to family, with an odds ratio of 0.93 (95% CI = 0.89–0.98, *p* = .006). Similarly, a higher level of secrecy to avoid stigma was associated with a decrease in the odds of comfort disclosing the diagnosis to family, with an odds ratio of 0.40 (95% CI = 0.17–0.98, *p* = .046). Using Nagelkerke’s pseudo-*R*^2^ coefficient, 28.8% of the variance in comfort with disclosure to family were explained by the overall model.

**Table 6.** Ordinal regression results for predictors of comfort with disclosure to family and friends for the combined sample (*N* = 80).

Variable	Estimate	*SE*	Wald	*p*	*OR*	95% CI	
						Lower	Upper
Comfort with disclosure to family							
Age	−0.07	0.03	7.70	.006	0.93	.89	0.98
Resources to cope	0.10	0.05	3.69	.055	1.11	1.00	1.23
Secrecy	−0.91	0.46	4.00	.046	0.40	0.17	0.98
Internalised shame	−0.18	0.10	3.08	.079	0.83	0.68	1.02
Nagelkerke *R*^2^ = .288							
Comfort with disclosure to friends							
Stigma-related harm	−0.03	0.03	.66	.418	0.97	0.92	1.04
Resources to cope	0.10	0.06	3.12	.077	1.11	0.99	1.24
Secrecy	−2.24	0.48	21.54	.000	0.11	0.04	0.27
Internalised shame	−0.08	0.10	0.67	.414	0.92	0.76	1.12
Nagelkerke *R*^2^ = .408							

*Note.* Nagelkerke *R*^2^ is a ‘pseudo’ *R*^2^ measure of variance accounted for by each model. It’s analogous to *R*^2^ in multiple regression, with a maximum value of 1. CI = confidence interval; OR = odds ratio; SE = standard error.

The ordinal regression model for comfort with disclosure to friends contained the predictors resources to cope with stigma, secrecy, internalised shame, and perceived stigma-related harm. Only secrecy was found to be a significant predictor; a higher level of secrecy was associated with a decrease in the odds of feeling comfortable disclosing to friends, with an odds ratio of 0.107 (95% CI = 0.04–.27, *p* = .000). The overall model explained 40.8% of the criterion variance using Nagelkerke’s pseudo-*R*^2^ coefficient.

## Discussion

This is, to our knowledge, the first study into the psychometric properties of scales measuring stigma-related constructs in a Dutch sample of people living with dementia and comparison of outcome measures of stigma between Dutch and English people living with dementia.

Analyses indicate that the stigma measures are reliable and valid for use in a Dutch population with high internal consistencies for most (sub)scales, as has previously been found for English versions of these scales (Bhatt et al., 2021). The only scale not showing adequate internal consistency was the internalised shame subscale of the Stigma Impact Scale, in line with previous studies with people living with dementia (Bhatt et al., 2021; Lion et al., 2020), perhaps partly due to the small number of items (*n* = 3). The study showed that Dutch participants who reported more severe stigma impact or consequences, i.e., social rejection, internalised shame, and social isolation, as well as perceived stigma-related harm and secrecy also reported lower self-esteem, whereas greater resources to cope with stigma were associated with higher self-esteem. This not only confirms the convergent validity of the Dutch versions of the scale, but also demonstrates the associations between perceived stigma, secrecy, and self-evaluation in people living with dementia. The positive correlation between internalised shame and secrecy is in line with the well-documented relationship between self-stigma and secrecy in other stigmatised populations (Chaudoir & Fisher, 2010; Corrigan et al., 2015; Paxton, 2002; Pescosolido & Martin, 2015) and people living with dementia in the UK (Bhatt et al., 2021). This may indicate that negative consequences can be felt even in the absence of overt stigma experiences. For example, secrecy may be associated with stigmatising self-appraisals rather than more overt forms of stigma such as social rejection or isolation. Measuring levels of secrecy, therefore, may be a way of operationalising internalised shame, especially given the greater psychometric robustness of the secrecy scale relative to the Stigma Impact Scale internalised shame subscale.

The Dutch-English comparison of stigma in people living with dementia builds on the recent survey conducted by Alzheimer’s Disease International (2019) and complements cross-cultural comparisons in the MEETINGDEM project (Lion et al., 2021; Szcześniak et al., 2021) and from members of the INTERDEM group for research on timely psychosocial interventions in dementia (Vernooij-Dassen et al., 2005). There was evidence of a small to moderate difference in the Stigma Impact Scale total score between the current Dutch and English samples, mainly due to more experienced social isolation in English people, which remained after controlling for differences in participant characteristics (i.e., age and time since diagnosis). The differences in social isolation may reflect different recruitment strategies, with Dutch participants recruited via the meeting centres programme. Although time since diagnosis accounted for some variance in the main effect of country and Stigma Impact Scale total score, a greater proportion was accounted for by age. Previous stigma research with people living with dementia has not controlled for age, that is, has not ruled out age as a contributing factor, but the current findings suggest that particular attention should be paid to understanding the role of age in the stigma impact, especially the stigma impact on younger people receiving a dementia diagnosis who often feel more isolated (Millenaar et al., 2016).

In line with previous disclosure research (Earnshaw et al., 2019; Henderson et al., 2017), we found that the majority of individuals would feel comfortable disclosing their dementia diagnosis to family and friends. This finding expands on earlier work that has emphasised the importance of confidants and social support in people living with a chronic condition or hidden disability (Camacho et al., 2020). It might therefore be valuable to explore the effects of perceived social support on the disclosure and stigma experiences of people living with dementia further. As expected, we also found that internal factors play a role in an individual’s disclosure comfort level: Higher levels of internalised shame and desire for secrecy were associated with lower disclosure comfort, whereas having resources to cope with stigma were associated with feeling more comfortable disclosing a dementia diagnosis to family and friends. These findings confirm the importance of dementia anti-stigma interventions and awareness campaigns (Ashworth, 2020; Mukadam & Livingston, 2012). However, given the cross-sectional design of this study, it is uncertain what the direction of the relationship of comfort with disclosure and the stigma-related variables is. More specifically, greater comfort with disclosure to social networks could be a coping resource that minimises secrecy and internalised shame, or less secrecy and internalised shame could enhance disclosure comfort.

Interestingly, our findings suggest that individuals who were older would feel less comfortable disclosing their diagnosis to their family. Though previous research has produced similar findings (Maguen et al., 2007), younger people living with dementia often also feel stigmatised and uncomfortable sharing the diagnosis because young-onset dementia is considerably less common (Millenaar et al., 2016). Our findings could be the result of the skewness of our data collected with the Comfort with Disclosure Scale. We therefore recommend reproducing this study with a larger, more age-diverse sample.

### Strengths and limitations

A strength of this study is the involvement of bilingual researchers, professional research translators, and use of the backward translation approach to maximise the likelihood of equivalence of meaning for each item in the translated questionnaires. However, there are also limitations to be considered. First and foremost, the study samples were small, which is a limitation especially given the need to adjust for demographic differences. The cross-cultural comparison should, therefore, be interpreted with caution. Preliminary psychometric properties can be explored with a small sample size (Johanson & Brooks, 2010), however, larger scale studies are necessary to confirm psychometric properties and country differences. Second, the majority of participants in this study identified as being from a White ethnic background. Due to the ethnic homogeneity, the results of our study may not be generalisable to individuals from other ethnic backgrounds. Future studies would benefit from providing a more diverse view of the stigma experiences of individuals living with dementia, especially since stigma may be experienced differently by individuals from other ethnic groups (Herrmann et al., 2018). A third limitation is the absence of data on educational level for the English sample, especially given the importance of educational level in previous studies of stigma impact in dementia (Lion et al., 2020). Finally, one of the difficulties in studies exploring disclosure is bias that can be created due to studies attracting participants that are generally more open about their diagnosis (Hilton et al., 2009; Pembroke et al., 2017). This may have been even more the case for the Dutch sample, which was predominantly recruited in Meeting Centres for people living with dementia and carers, and the English sample that was drawn from the Join Dementia Research database. In the absence of comparative data, we are unable to say whether the results of this study also apply to people living with dementia who actively conceal their diagnosis from others.

## Implications

The present study presents a starting point for understanding the relationship between stigma and comfort with diagnosis disclosure in people living with dementia, a concept that has not previously been investigated in dementia research. Stigma affects people living with dementia worldwide and concealing the diagnosis can have negative implications such as delayed help-seeking (Alzheimer’s Disease International, 2019). More initiatives should be undertaken to create a dementia-friendly society and neighbourhoods in which people living with dementia feel socially included and accepted; thus, preventing feelings of social isolation and rejection. In addition, how stigma affects individuals living with dementia can also vary depending on their cultural background (Herrmann et al., 2018). We therefore encourage a better understanding of stigma and its manifestation within different cultures.

Understanding and measuring the way in which people living with dementia feel about disclosing their diagnosis to their social networks as well as how stigma shapes their experiences can be a means of tailoring support plans, resources, and clinical interventions. It is necessary for stigma to be considered during clinical interviews and assessments to ascertain the impact of stigma on individuals.

## Conclusions

This study has shown that the Dutch versions of the three stigma scales are reliable and valid measures in a population of people living with dementia. Cross-cultural differences in stigma experience of people living with dementia of different ages require further investigation. The study contributes to research on disclosure decisions and stigma experienced by people living with dementia by confirming that high levels of disclosure comfort are common and that stigma experiences play an important role in disclosure comfort, even in the absence of overt stigma.
